# Education protects against coronary heart disease and stroke independently of cognitive function: evidence from Mendelian randomization

**DOI:** 10.1093/ije/dyz200

**Published:** 2019-09-28

**Authors:** Dipender Gill, Anthoula Efstathiadou, Kristopher Cawood, Ioanna Tzoulaki, Abbas Dehghan

**Affiliations:** 1 Department of Epidemiology and Biostatistics, School of Public Health, Imperial College London, London, UK; 2 Her Majesty’s Treasury, London, UK; 3 Medical Research Council–Public Health England Centre for Environment, School of Public Health, Imperial College London, London, UK; 4 Department of Hygiene and Epidemiology, University of Ioannina Medical School, Ioannina, Greece

**Keywords:** Mendelian randomization, education, cognition, coronary heart disease, stroke

## Abstract

**Background:**

There is evidence that education protects against cardiovascular disease. However, it is not known whether such an effect is independent of cognition.

**Methods:**

We performed two-sample Mendelian randomization (MR) analyses to investigate the effect of education and cognition, respectively, on risk of CHD and ischaemic stroke. Additionally, we used multivariable MR to adjust for the effects of cognition and education in the respective analyses to measure the effects of these traits independently of each other.

**Results:**

In unadjusted MR, there was evidence that education is causally associated with both CHD and stroke risk [CHD: odds ratio (OR) 0.65 per 1-standard deviation (SD; 3.6 years) increase in education; 95% confidence interval (CI) 0.61–0.70, stroke: OR 0.77; 95% CI 0.69–0.86]. This effect persisted after adjusting for cognition in multivariable MR (CHD: OR 0.76; 95% CI 0.65–0.89, stroke OR 0.74; 95% CI 0.59–0.92). Cognition had an apparent effect on CHD risk in unadjusted MR (OR per 1-SD increase 0.80; 95% CI 0.74–0.85), however after adjusting for education this was no longer observed (OR 1.03; 95% CI 0.86–1.25). Cognition did not have any notable effect on the risk of developing ischaemic stroke, with (OR 0.97; 95% CI 0.87–1.08) or without adjustment for education (OR 1.04; 95% CI 0.79–1.36).

**Conclusions:**

This study provides evidence to support that education protects against CHD and ischaemic stroke risk independently of cognition, but does not provide evidence to support that cognition protects against CHD and stroke risk independently of education. These findings could have implications for education and health policy.

**Key Messages**

- This work applied the multivariable Mendelian randomization technique to study the effects of educational attainment and cognitive function independently of each other on risk of coronary heart disease and ischaemic stroke, respectively.
- We found that educational attainment affects risk of coronary heart disease and ischaemic stroke independently of cognitive function, but did not identify an effect of cognitive function on these outcomes that was independent of educational attainment.
- These results are in keeping with previous work in this area and add to the body of evidence now available to inform public health and educational policy.

## Introduction

Coronary heart disease (CHD) and stroke together make up the largest cause of morbidity and mortality worldwide, accounting for over 15 million deaths in 2016.^1^ Higher cognitive performance and longer duration of education are closely related traits,^2^^,^^3^ and have both been inversely associated with the risk of CHD and stroke in observational studies.^4^^,^^5^ However, such associations can be affected by confounding from unmeasured or unknown factors, and are therefore not reliable for inferring causality.^6^ Randomized controlled trials (RCTs), on the other hand, are not a plausible option for studying the effects of cognition or education.^7^ Whereas previous work has investigated the effect of educational attainment on cardiovascular disease risk^8^^,^^9^ further disentangling the independent effects of education and cognition would have important implications for public health and educational policy, particularly with regard to allocation of resources towards targeting the relevant exposure. For example, in the scenario where education is causally related to developing CHD or stroke independently of cognitive function, but not vice versa, strategies for increasing education rather than cognitive ability would better protect against adverse health outcomes.

Mendelian randomization (MR) is an instrumental variable method that can overcome some of the limitations of observational studies, by using genetic variants as instruments to study the effect of varying an exposure. The random allocation of such variants thus avoids the effect of confounding environmental factors to make causal inferences on an outcome of interest.^10^ Furthermore, the presence of genetic variants from conception also overcomes the potential reverse causation bias that can limit the interpretation of traditional observational analyses. However, a possible source of bias in MR relates to pleiotropy of the genetic instruments used, where they affect the outcome through pathways at least partly independent of the exposure, to violate the requisite assumptions of this model.^10^

An extension of the MR approach is multivariable MR (MVMR),^11^ which additionally allows adjustment for pleiotropic effects of the instruments through known pathways for which genetic association estimates are also available.^12^ This is particularly useful when the exposures of interest are related, as is the case for educational attainment and cognitive function, which have a high degree of phenotypic and genetic correlation.^2^^,^^3^ Previous work has used MR to provide evidence of a bidirectional relationship between educational attainment and cognitive performance,^13^ and furthermore it is plausible that educational attainment and cognitive function might mediate some of each other’s respective effects on cardiovascular disease outcomes, as well as acting as potential confounders.^14^ Given the importance of establishing understanding of the independent effects of these traits for implementing policies to reduce cardiovascular disease, in this study we performed MR analyses to investigate the total and independent (i.e. direct) effects of educational attainment and cognitive function on risk of CHD and ischaemic stroke, respectively.^12^ Specifically, we used conventional (univariable) MR to estimate the total effects of educational attainment and cognitive function (i.e. encompassing any mediation or genetic confounding from each other) on CHD and ischaemic stroke risk, respectively, and MVMR to investigate their direct effects (i.e. excluding any mediation or genetic confounding from each other).^12^^,^^15^

## Methods

### Genetic association estimates

Association estimates between single-nucleotide polymorphisms (SNPs) and educational attainment were derived from the publicly available summary data of a genome-wide association study (GWAS) meta-analysis (that excluded the 23andMe cohort) of 766 345 individuals of European ancestry.^2^ Instruments were selected based on their genome wide-significance (*P*-value <5 × 10^–8^) and independence (linkage disequilibrium r^2^≤0.1). Educational attainment was measured as the number of years of schooling that individuals completed. Due to discrepancies in educational systems and qualifications between the cohorts for educational attainment, the International Standard Classification of Education (ISCED) system was used to match educational qualifications across countries into one of seven harmonized ISCED categories. Supplementary Table 1, available as Supplementary data at *IJE* online, details how ISCED scores were mapped to years spent in education.^3^ Estimates are presented in standard deviation (SD) units, with 1-SD corresponding to 3.6 years of education.

For cognitive function, SNPs were taken from a GWAS meta-analysis performed in the UK Biobank and COGENT consortium in 257 841 participants of European ancestry.^2^ Instruments were selected using the same criteria as for educational attainment. Cognitive performance was evaluated in UK Biobank using a test of verbal-numerical reasoning,^16^ which consisted of 13 questions designed to assess verbal and mathematical ability and which correlated highly with other measures of intelligence.^17^^,^^18^ Various neuropsychological tests were used to measure cognitive function in the COGENT study.^19^^,^^20^ Each of the 35 COGENT sub-studies administered a mean of eight (SD = 4) neuropsychological tests, with each included participant required to have data available from at least three domains of cognitive function.^19^ The most commonly administered tests in the COGENT study assessed digit span, digit symbol coding, phonemic fluency, semantic fluency, trail-making, verbal memory for stories, verbal memory for words, visual memory, vocabulary and word reading.^19^ Genetic association estimates are presented in SD units. F statistic and R^2^ values were estimated for the instrument SNPs of both traits to evaluate their strength in conventional MR and proportion of phenotypic variance explained, respectively (with the specific formulae used detailed in Supplementary Tables 2 and 3, available as Supplementary data at *IJE* online).^21^^,^^22^ Instrument SNPs were not pruned based on any secondary genetic associations or the relative strengths of their associations with educational attainment or cognitive function.

The CARDIoGRAMplusC4D 1000 Genomes-based GWAS meta-analysis was used to obtain genetic association estimates for CHD.^23^ There were 60 801 cases and 123 504 controls, with the majority of participants of European ancestry and a full breakdown of ethnic groups provided in Supplementary Table 4, available as Supplementary data at *IJE* online. Adjustment was made for genetic ancestry using the genomic control method, and the CHD definition was broad, including acute coronary syndrome and angina.^23^

For ischaemic stroke, instrument SNP genetic association estimates were extracted from a GWAS of 37 792 cases and 397 209 controls performed by the National Institute of Neurological Disorders and Stroke (NINDS) Stroke Genetics Network (SiGN), and were downloaded from the Cerebrovascular Disease Knowledge Portal.^24^^,^^25^ Participants were of mostly of European ancestry, but also included individuals of Hispanic and African origin, with a full breakdown of proportions provided in Supplementary Table 5, available as Supplementary data at *IJE* online. Adjustment was made for genetic ancestry using principal component analysis,^24^ and the ischaemic stroke definition was based on a classic definition given by the World Health Organization: rapidly developing clinical signs of focal (or global) disturbance of cerebral function, with symptoms lasting 24 h or longer or leading to death, with no apparent cause other than of vascular origin.^26^

Cohort details for all participating studies in these four GWAS meta-analyses are provided in Supplementary Table 6, available as Supplementary data at *IJE* online. Overlap in the cohorts used for the exposure and outcome genetic association estimates is also detailed in Supplementary Table 6, as this can have implications for bias in MR analysis.^27^

### Mendelian randomization analyses

We performed power calculations using the mRnd power calculator for conventional MR, available at [http://cnsgenomics.com/shiny/mRnd/].^28^ We estimated the smallest detectable protective effect of education and cognitive function on CHD and ischaemic stroke risk, respectively, required to achieve 80% statistical power, given the available sample sizes and phenotypic variance explained by the instruments.

Conventional (unadjusted) MR analyses examining the total effects of educational attainment and cognitive function, respectively, separately on CHD and ischaemic stroke, were performed. Specifically, we derived MR estimates for individual SNPs using the Wald ratio, with standard errors calculated using second-order weights to account for possible measurement error in both exposure and outcome association estimates.^29^ Fixed-effects inverse variance weighting (IVW) was then used to pool results across instrument SNPs for a particular exposure and thus maximize statistical power.^30^ In secondary analyses, we also performed the (random-effects) MR-Egger and weighted median statistical sensitivity analyses to investigate whether similar MR estimates were obtained under these models that relax their requisite assumptions on the presence of pleiotropic genetic variants that affect risk of the considered outcome independently of the exposure under study.^31^^,^^32^ MR-Egger performs a regression of the SNP-outcome association estimates conditioned on the SNP-exposure outcome association estimates weighted for the precision of the SNP-outcome association estimates to generate pleiotropy-adjusted MR estimates, with a non-zero intercept serving as a test for directional pleiotropy.^31^ MR-Egger requires that the strength of the instruments (i.e. the SNP-exposure associations) are not correlated to any direct (independent of the exposure) effect that they have on the outcome, and can produce biased estimates if this assumption is violated.^31^ The weighted median approach orders the MR estimates produced by individual instrument SNPs by their magnitude weighted for their precision, and selects the median result as the overall MR estimate, with confidence intervals calculated by bootstrapping.^32^ Weighted median MR is generally robust when more than half of the information for the analysis comes from valid instruments.^32^

To investigate the independent effects of educational attainment and cognitive function on CHD and ischaemic stroke risk, we performed regression-based MVMR using summary data, with adjustment made for the genetic associations of the educational attainment instruments for cognitive function, and vice versa.^11^^,^^33^ Specifically, the same instrument SNPs and genetic association estimates were used as in the non-multivariable (unadjusted) MR analyses detailed above. However, adjustment was made for association of the instruments SNPs with cognitive performance when considering educational attainment as an exposure. Similarly, adjustment was made for association of the instruments SNPs with educational attainment when considering cognitive performance as an exposure. The conventional summary data regression-based method was used for this, which performs a linear regression of the SNP-outcome genetic association estimate against the SNP-exposure association estimate and the SNP-genetic confounder association estimate, weighted for the inverse standard error of the SNP-outcome estimate.^11^^,^^33^ The intercept is fixed at zero, and there is no interaction term.^11^^,^^33^

All assumptions made for a conventional MR analysis also apply in this multivariable model, including that the instruments must be strongly associated with the exposures of interest, must be associated with the outcome only through the included exposures or genetic confounders (and not via any other pathway) and must be independent of confounders that influence the exposure-outcome relationship.^11^ Any pleiotropic association of the instruments with CHD or ischaemic stroke risk through pathways independent of both educational attainment and cognitive function would thus result in bias of these final MVMR estimates. To investigate this possibility, we additionally performed (random-effects) MVMR-Egger, which does not fix the intercept of the MVMR regression to zero, but instead uses this as a test for directional pleiotropy and generates pleiotropy-adjusted effect estimates.^34^ Furthermore, we also performed an MVMR median regression sensitivity analysis, which estimates the median of the SNP-outcome genetic association estimate conditional on the SNP-exposure association estimate and the SNP-genetic confounder association estimate, with the intercept set to zero and weighted for the inverse standard error of the SNP-outcome estimate. Standard errors for MVMR median regression were estimated by bootstrapping. This approach is similar to the conventional regression-based MVMR method that we use, except that it estimates the median of the SNP-outcome association estimate rather than its mean. To further explore the possibility that the findings of our MVMR analyses are related to particular pleiotropic variants, we repeated all analyses 1000 times after randomly sampling (without replacement) a subsample of only 200 of the available instrument SNPs, separately for both educational attainment and cognitive function, and investigating the distribution of MR estimates. This approach also allowed us to investigate whether any discrepancy in the findings for educational attainment or cognitive function might relate to the different number of instrument SNPs available. In all MR analyses, the effect alleles for genetic association estimates were aligned to represent an increase in the primary exposure under investigation.

All analyses were performed using R (version 3.4.2). The TwoSampleMR R package was used to perform MR-Egger and weighted median MR, and for clumping SNPs.^35^ In all MR analyses, harmonization of SNP genetic association estimates from different studies was performed by aligning effect alleles. The data used in this work are publicly available summary results from published GWAS meta-analyses, for which ethical approval and patient consent were obtained in the original cited studies. All data used in this study can additionally be obtained from the corresponding author upon reasonable request.

## Results

In total, there were 625 instrument SNPs (*P*-value <5 × 10^–8^ and r^2^≤0.1, Supplementary Table 2, available as Supplementary data at *IJE* online) for educational attainment and 226 instrument SNPs (*P*-value <5 × 10^–8^ and r^2^≤0.1, Supplementary Table 3, available as Supplementary data at *IJE* online) for cognitive function. Conventional (for IVW MR) F statistic values for individual instrument SNPs ranged from 30 to 240 (Supplementary Tables 2 and 3), and combined were 27 596 for educational attainment and 9420 for cognitive function, with means of 44 and 42, respectively. Overall, the 625 instrument SNPs for educational attainment explained 3.6% of the variability in education and the 226 instrument SNPs for cognitive performance explained 3.7% in cognition. Power calculations for the conventional IVW MR analyses indicated greater than 80% statistical power to detect an odds ratio per 1-SD increase in the considered exposure smaller than 0.92 for all four analyses.^28^

**Figure 1.**
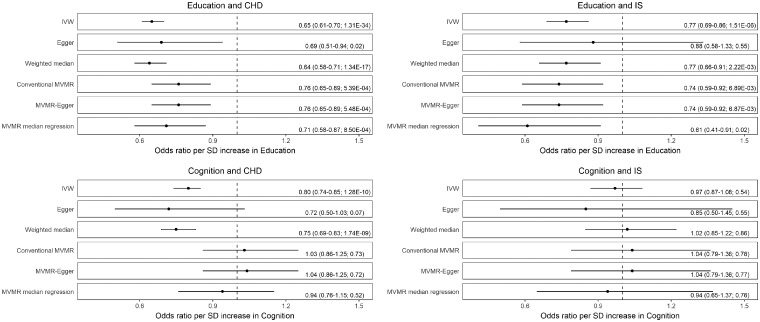
Mendelian randomization analysis results. When education is the exposure, multivariable Mendelian randomization (MVMR) analyses adjust for cognition. When cognition is the exposure, MVMR analyses adjust for education. Odds ratios are listed, with 95% confidence intervals and *P*-values in brackets. CHD, coronary heart disease; IS: ischaemic stroke, SD, standard deviation.

The results of the MR analyses are summarized in Figure 1 (and Supplementary Table 7, available as Supplementary data at *IJE* online). All analyses showed a consistent protective effect of educational attainment on risk of CHD and ischaemic stroke, irrespective of whether adjustment was made for cognitive function. Conversely, cognitive function showed a protective effect on risk of CHD but not of ischaemic stroke when not adjusted for educational attainment. This protective effect of cognitive function on CHD risk was not observed after adjusting for educational attainment in MVMR approaches. The MR-Egger and MVMR-Egger intercepts were not suggestive of directional pleiotropy in any analysis (Supplementary Table 8, available as Supplementary data at *IJE* online).

**Figure 2.**
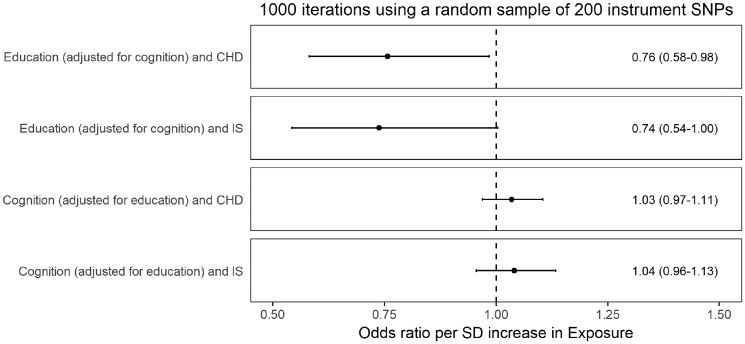
Results of the main regression-based multivariable Mendelian randomization analyses when performed 1000 times randomly sampling 200 instruments SNPs from the available pool of 625 for educational attainment and 226 for cognitive function. The mean odds ratios are displayed, with 95% confidence intervals in brackets. CHD, coronary heart disease; IS, ischaemic stroke; SD, standard deviation; SNP, single-nucleotide polymorphism.

Randomly selecting 200 instrument SNPs from the total available pool of 625 for educational attainment and 226 for cognitive function, respectively, and repeating the conventional MVMR analyses 1000 times (Figure 2, and Supplementary Figures 1–4, available as Supplementary data at *IJE* online) produced similar results to the conventional MVMR analyses using all instrument SNPs that are presented in Figure 1.

## Discussion

Our unadjusted MR analyses supported that educational attainment has a protective effect on both CHD and ischaemic stroke, and further that cognitive function also has a protective effect on CHD risk. The wider confidence intervals of the MR-Egger analysis may be related to the lower statistical power of this approach. The MVMR approaches used in this work went further to support that educational attainment has a protective effect on CHD and ischaemic stroke risk independent of cognitive function, but did not support an effect of cognitive function on CHD risk independent of educational attainment. Similar results were obtained when using statistical methods more robust to the inclusion of pleiotropic variants, suggesting that potential genetic associations with the outcome independent of the exposure, or with confounders of the exposure-outcome association, were unlikely to be introducing bias that would affect our conclusions. Similar results were also obtained when randomly sampling 200 instrument SNPs for the MVMR analyses, suggesting that the findings were not attributable to a discrepancy in the number of available instrument SNPs for the different analyses.

The protective role of educational attainment on cardiovascular disease (CVD) has been described in previous observational work,^5^^,^^36^^,^^37^ as well as recent MR studies,^8^^,^^9^ but adjustment for cognitive function was not performed in these particular analyses and neither did they focus on the distinction between educational attainment and cognitive function in relation to cardiovascular risk. However, a recent study has used MVMR to suggest that the protective effect of education on likelihood of smoking was not due to an effect of cognitive function,^14^ and as smoking is an established risk factor for cardiovascular disease, this is consistent with our current work. The attenuation of cognitive function’s effect on CHD risk after adjusting for education has been a recurrent finding in observational studies.^4^^,^^38^^,^^39^ This was not the case for the cognition-stroke relationship, as cognition was described to be an independent predictor of stroke irrespective of education, in previous observational work.^40^^,^^41^ However, a variety of tests for measuring cognitive function were used, with not all of them resulting in consistent findings. The latter perhaps relates to the various domains that fall under the term ‘cognitive function’ and the need to distinguish between these more precisely in further work.

The explanation for why educational attainment protects against CVD independently of cognitive function but not vice versa may relate to education’s broad benefits. Higher educational attainment is associated with a healthier lifestyle, an occupation with safer working conditions and better access to health care.^42–44^ Highly educated individuals tend to avoid major CVD risk factors such as smoking and excessive alcohol intake, and are generally informed of their harmful effects on health.^45^^,^^46^ Recent MR and observational study has suggested that approximately 40% of the protective effects of education on cardiovascular disease risk may be mediated through more favourable profiles for blood pressure, body habitus and smoking behaviour, although this work did not consider the role of cognitive function.^47^ The positive effects of educational attainment in CVD prevention may also be through developing healthier habits, such as exercise and diet, which can last into adulthood.^5^ Furthermore, income among people who spend a greater number of years in education is higher,^48^ which in turn may result in an improved lifestyle and lower levels of stress.^49^ Cognitive function likely plays a secondary role, as the reported beneficial effects of higher cognition on many health outcomes, including maintenance of blood pressure and body weight within healthy limits, appear to be mediated by education.^50^^,^^51^ This suggests that it is the additional skills, behaviours and quality of life brought about by greater educational attainment, rather than higher intelligence on its own, that protect from outcomes such as CVD.

Education and cognition have a close relationship, with evidence supporting bi-directional effects.^13^ Individuals with higher cognitive ability tend to spend more time in education,^52^ and higher educational attainment can improve cognitive performance.^53^^,^^54^ Therefore, our findings offer an important contribution towards understanding which has independent causative effects on health outcomes, especially when considering policies for population health optimization. Our battery of MR analyses (Figure 1, Supplementary Table 7, available as Supplementary data at *IJE* online) all produced consistent results to suggest that isolated pleiotropic SNPs were unlikely to be responsible for our conclusions. Whereas educational attainment and cognitive function may have a shared aetiology and bidirectional mediating effects, the MVMR approaches that we applied were able to disentangle their direct effects and thus offer important insight in terms of disease prevention,^12^ particularly as education is a modifiable factor. Therefore an increase in the mandatory years of education may, for example, have a protective effect on risk of cardiovascular disease, independently of whether cognitive function is also increased. Such interventions regarding education policy have previously lowered morbidity and mortality from many chronic diseases including CHD and stroke.^55^ The UK’s recent increase in the age of mandatory education, from 16 to 18 years, represents just such an example.^55^ Any potential differential impact of such policy on health outcomes will not be wholly apparent for many years. Of relevance, our study does not inform on whether similar effects would be observed if education were to take a different form, such as through work-based training rather than a traditional academic programme.

In this study, we performed MVMR alongside conventional, unadjusted MR to investigate the effects of education and cognition on CHD and stroke risk, independently of each other. However, both MVMR and MR can only be valid tools for inferring causality when the requisite assumptions are held.^30^^,^^33^ Although the instruments used for the conventional MR were all strong (with F statistics for individual variants all greater than 30), there is no currently available method to estimate instrument strength in MVMR when using summary data alone.^33^ Therefore, this makes it impossible to assess for possible bias relating to the use of weak instruments in our MVMR analysis.^33^ Directional pleiotropy can be a source of bias in MR analyses, and reassuringly this seemed unlikely in our work because the statistical sensitivity analyses that relax their assumptions of the presence of pleiotropic variants also found similar results, although with the notably wide 95% CIs of the MR-Egger approach that are likely related to low statistical power (Figure 1). However, results from both the MR-Egger and MVMR-Egger are unbiased only when the ‘instrument strength independent of direct effect’ or ‘InSIDE’ assumption holds. The latter requires that the genetic associations of the instruments with the exposure are independent of their direct effects on the outcome,^31^ an assumption that cannot be tested.^56^ In our current study, it may well be that genetic associations of instrument SNPs for educational attainment and cognitive function are correlated to their direct effect on CHD or ischaemic stroke risk, thus violating InSIDE.

A large proportion of the population used to obtain genetic association estimates for educational attainment (58%) and cognitive function (86%) were part of UK Biobank.^2^ This represents a select cohort that may not be representative of the wider general population,^57^ and indeed such selection has been shown to potentially introduce bias into MR analysis.^58^ Similarly, another possible limitation could be related to participant overlap in the GWAS meta-analyses for education and cognitive ability with those for CHD and ischaemic stroke (Supplementary Table 6, available as Supplementary data at *IJE* online). This situation could theoretically introduce bias in the consequent MR analysis,^27^ although from details on the cohorts used for each study (Supplementary Table 6), it is unlikely that any such overlap in the exposure and outcome populations occurred for more than 10% of participants. Furthermore, two precautions against this were taken: strong instruments that are less susceptible to such bias were used,^59^ and the analysis was performed using second-order weights, which decreases false-positive findings as compared with using first-order weights when using partially overlapping datasets (at the cost of potentially decreasing the power to detect heterogeneity between MR estimates produced by different instrument SNPs).^29^ Genetic ancestry is an important confounder to be considered while applying MR, for example, because some of the variants may be absent or have substantial difference in allele frequency or effect size across populations with different genetic ancestries. The European participants used in the educational attainment and cognitive function GWAS meta-analyses contrasted with the multi-ethnic populations used for study of CHD and ischaemic stroke (Supplementary Tables 4 and 5, available as Supplementary data at *IJE* online), thus introducing a potential source of bias. However, over 75% of these multi-ethnic studies were still made up of European-ancestry participants, and thus the analysis remained centred on this ethnic group. Considering the GWAS meta-analyses used to obtain genetic associations for educational attainment and cognitive function, it is likely that estimates were inflated because of parental rearing effects affecting these traits independently of inherited genetic variants.^2^ This would potentially overestimate the SNP-exposure estimates and create bias towards the null hypothesis in the context of two-sample MR. Furthermore, the associations of the genetic variants with exposures and outcomes may vary depending on the particular environmental context,^2^ potentially introducing a further source of bias into our MR analysis.

Finally, in our study education was evaluated as the number of years an individual has spent at an academic institution. Education, however, is rather a process of learning and growth that may not necessarily be confined to such definitions. Obtaining related skills through alternative means may also be of relevance and requires further study. Similarly, cognitive function has various domains.^18^^,^^19^ Verbal-numerical attainment and neuropsychological tests were used to quantify this in the genetic association estimates that we used,^2^ but this may represent an amalgamation of various component traits that each have distinct effects on cardiovascular disease risk. To this end, further work is required to disentangle the relationship between different cognitive domains and clinical outcomes.

In conclusion, our study provides evidence supporting a causal effect of education on the risk of developing CHD and stroke, independently of cognitive function. In contrast, we did not find evidence to support that cognitive function affects CHD and stroke risk independently of educational attainment. These results are in keeping with previous work in this area, and add to the body of evidence now available to inform public health and educational policy.

Summary genetic data for both cognitive function and educational attainment can be downloaded from the Social Science Genetic Association Consortium (SSGAC) portal [https://www.thessgac.org/data] (Lee *et al*.).^2^ Summary genetic data for coronary heart disease can be downloaded from CARDIoGRAMplusC4D Consortium portal [http://www.cardiogramplusc4d.org/data-downloads/] [CARDIoGRAMplusC4D 1000 Genomes-based GWAS, additive model (as in this analysis an additive genetic model is assumed)].^23^ Summary genetic data for ischaemic stroke can be downloaded from the cerebrovascular disease knowledge portal [http://cerebrovascularportal.org/informational/downloads] [loci associated with ischaemic stroke and its subtypes (SiGN): a genome-wide association study].^24^^,^^25^

## Funding

D.G. is funded by the Wellcome 4i programme at Imperial College London.

## Supplementary Material

dyz200_Supplementary_Data
