# Supplementary material for: Education protects against coronary heart disease and stroke independently of cognitive function: evidence from Mendelian randomization
Source: Int J Epidemiol. 2019 Sep 28;48(5):1468–77. doi: 10.1093/ije/dyz200 (PMC6857750; doi:10.1093/ije/dyz200)

**Supplementary Figures**

Supplementary Figure 1. A histogram depicting results of the main regression-based multivariable Mendelian randomization analysis investigating the effect of educational attainment (adjusted for cognitive function) on coronary heart disease risk, when performed 1000 times randomly sampling 200 instruments SNPs from the available pool of 625. CHD: coronary heart disease; SD: standard deviation.


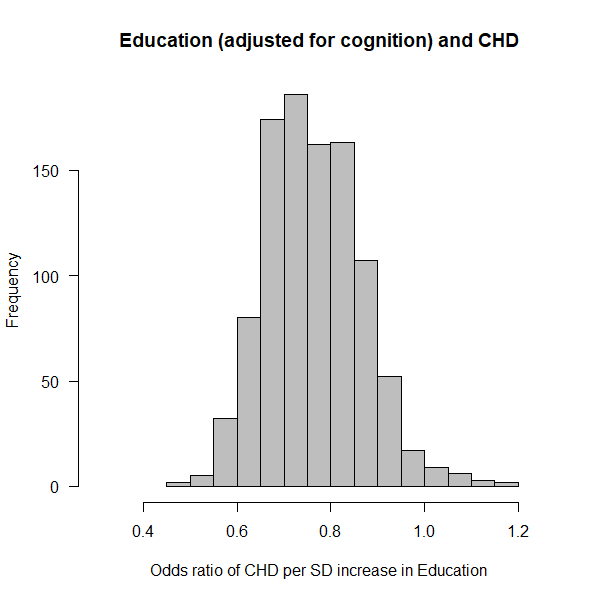


Supplementary Figure 2. A histogram depicting results of the main regression-based multivariable Mendelian randomization analysis investigating the effect of educational attainment (adjusted for cognitive function) on ischemic stroke risk, when performed 1000 times randomly sampling 200 instruments SNPs from the available pool of 625. IS: ischemic stroke; SD: standard deviation.


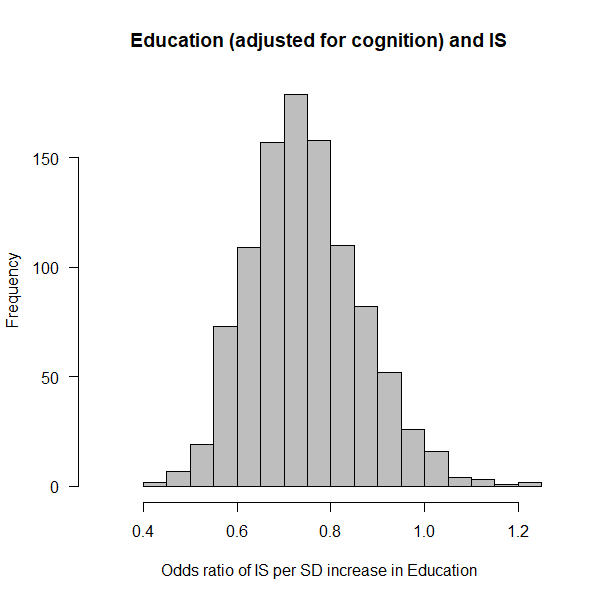


Supplementary Figure 3. A histogram depicting results of the main regression-based multivariable Mendelian randomization analysis investigating the effect of cognitive function (adjusted for educational attainment) on coronary heart disease risk, when performed 1000 times randomly sampling 200 instruments SNPs from the available pool of 226. CHD: coronary heart disease; SD: standard deviation.


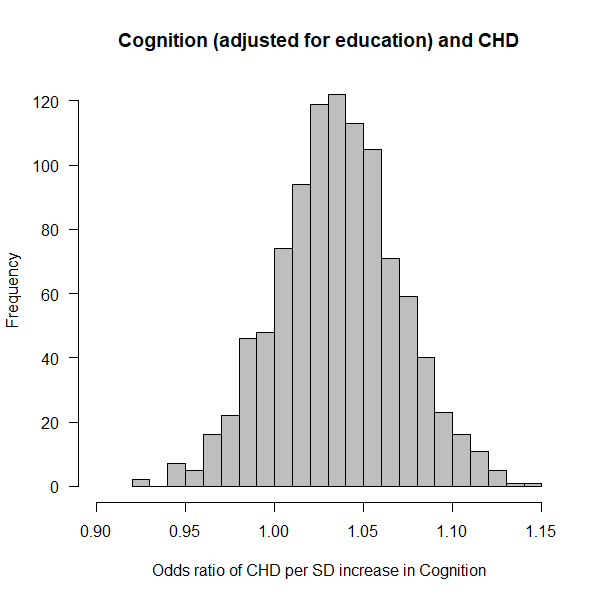


Supplementary Figure 4. A histogram depicting results of the main regression-based multivariable Mendelian randomization analysis investigating the effect of cognitive function (adjusted for educational attainment) on ischemic stroke risk, when performed 1000 times randomly sampling 200 instruments SNPs from the available pool of 226. IS: ischemic stroke; SD: standard deviation.


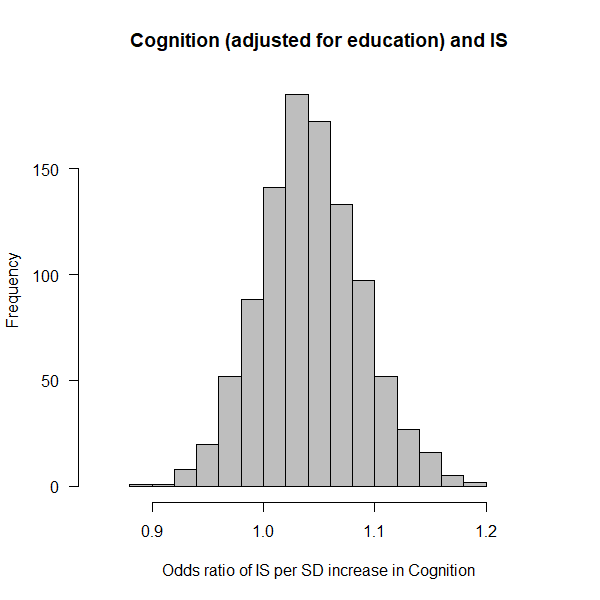

Supplement: dyz200_Supplementary_Data [file dyz200_supplementary_data.zip › dyz200-Suppl_data/Supplementary_Figures.docx]
